# A Pilot Study of a Psychoeducational Group Intervention Delivered at Asylum Accommodation Centers—A Mixed Methods Approach

**DOI:** 10.3390/ijerph17238953

**Published:** 2020-12-01

**Authors:** Anna Leiler, Elisabet Wasteson, Joanna Holmberg, Anna Bjärtå

**Affiliations:** 1 Institution of Psychology and Social Work, Mid Sweden University, 831 25 Östersund, Sweden; elisabet.wasteson@miun.se (E.W.); holmberg_joanna@hotmail.com (J.H.); anna.bjarta@miun.se (A.B.); 2 Region Örebro County, 703 54 Örebro, Sweden

**Keywords:** asylum seekers, mental health, pilot study, psychoeducation, psychosocial intervention

## Abstract

Asylum seekers suffer high levels of distress but have restricted access to mental health care. This paper constitutes an evaluation of a psycho-educational group intervention, called AMIN, which was provided at two asylum accommodation centers in Sweden. A mixed-methods approach was used. To assess potential effectiveness, acceptability, and feasibility, quantitative outcome measures were combined with qualitative information from interviews with both intervention participants and staff providing the intervention. Potential effectiveness in reducing symptoms of distress and insomnia and in increasing physical quality of life was found, even though the intervention participants suffered from more severe distress than expected. In general, the intervention seemed to be acceptable to both participants and staff, with concrete strategies seeming more meaningful than abstract psychological techniques. Finally, regardless of the asylum process itself being a complicating factor, the intervention seemed feasible to deliver to individuals with different backgrounds and conditions. Taken together, these results indicate that some sessions may need further elaboration, but also that the transition to a randomized control trial is reasonable.

## 1. Introduction

The time spent in an asylum accommodation center after arriving in a new country contains many challenges. Many individuals who have fled their homes have experienced potentially traumatic events both before and after leaving their homes [1,2]. Living in an asylum accommodation center often implies overcrowding, passivity, and isolation from the surrounding society, and recent reports show that individuals residing in asylum accommodation centers show high levels of distress [3,4]. Although the need for psychosocial interventions is clear, asylum seekers have limited access to mental health care [5].

To increase access to care, a stepped-care model, as described in the Inter-Agency Standing Committee (IASC) guidelines on mental health in emergency settings [6], could be applied. While only a small percentage of the survivors of an emergency is expected to be in need of specialized psychiatric care, a larger group can be expected to be in need of basic psychosocial support. According to the IASC, support at this level can be provided by trained and supervised workers without a formal education in psychiatry. In line with this, several interventions that can be delivered by laypersons have been developed and used with refugee populations in high-income countries (see, for example, Problem Management Plus PM + [7], and Teaching Recovery Techniques, TRT [8]).

In the project Assessment of Mental Health and Early Intervention for Refugees (AMIR), a brief, psycho-educational group intervention, called the Amir Intervention (AMIN), “amin” also meaning “trustworthy” in both Arabic and Dari, was developed. AMIN was created as a response to the fact that asylum seekers show high levels of distress but experience barriers in accessing mental health care. The intervention was developed with the aim of being feasible to deliver for any health care personnel after a brief training, and designed to be delivered in settings such as asylum accommodation centers. Due to the lack of a controlled situation, highly controlled studies with asylum seekers are rare. The inclusion of qualitative evaluations has been suggested to increase understanding of intervention research [9,10,11]. Listening to the voices of the asylum seekers could help ensure that an intervention is tailored to their specific needs. Since expressions and perceptions of mental health problems vary between cultures [12], a qualitative approach can further be useful in assessing whether or not a new intervention is meaningful and acceptable to the target group. To increase understanding of factors that may complicate or facilitate intervention delivery in the midst of the complex asylum process, the providers’ perspectives are important to include.

Using both quantitative outcome measures and interviews with participants and staff, this study aims to provide a preliminary evaluation of the intervention AMIN. Specifically, we addressed the following issues:

- Does the intervention hold potential effectiveness in reducing symptoms of distress, insomnia, and symptom catastrophizing, and in increasing self-perceived health and quality of life?
- Is the intervention perceived as acceptable by intervention participants and staff?
- Is the intervention feasible to deliver, with regard to the difficult circumstances of the asylum process?

Although the Consolidated Standards of Reporting Trials (CONSORT) extension to randomized pilot and feasibility trials [13] does not directly apply to nonrandomized pilot studies, the recommendations were followed when applicable.

## 2. Materials and Methods

### 2.1. Design

A mixed-methods design was used, in which interviews were embedded within a pretest–posttest intervention trial [14]. Quantitative data were collected pre and post the intervention. Qualitative interviews, with both intervention participants and staff, were embedded after the intervention, with the purpose of assessing both the participants’ and the staff’s perceptions of the intervention. The qualitative results were integrated with the quantitative outcomes to provide insights on potential effectiveness, acceptability, and feasibility. The study was approved by the regional ethical board (DNR 2017/320-32).

### 2.2. Participants

#### 2.2.1. Quantitative Sub-Study

To be able to participate, the individual had to be over the age of 18, speak either Dari or Arabic, and live in the asylum accommodation center in question. No other inclusion or exclusion criteria were applied. In total, 52 participants attended an information session in which they consented to participate in the study and completed the pre-assessment. Data from ten individuals were lost since the correct code failed to be registered for these participants. Another 17 participants dropped out for unknown reasons. The remaining 25 individuals constitute the final sample for the quantitative evaluation. The participants’ mean age was 36.1 (*SD* = 11.2). Eighteen of the participants (72%) were men, and seven (28%) were women. Seventeen (68%) participants spoke Dari, and eight (32%) spoke Arabic. Twenty-two (88%) participants had not received a residence permit while entering the intervention, whereas three (12%) participants had. Most participants in the Dari groups came from Afghanistan. The participants in the Arabic speaking intervention group came from several different countries in the Middle East. To retain anonymity, nationality is not presented.

#### 2.2.2. Qualitative Sub-Studies

##### Intervention Participant Interviews

About one month after finishing the Dari speaking intervention groups at the second center, we started contacting all Dari-speaking intervention participants at this site, from whom we had pre- and post-quantitative assessments. We managed to reach five of them, of which all consented to participate in the qualitative evaluation, constituting the final sample for the intervention participant interviews. Two of them were female, and three were male. Since the first accommodation center had been closed down, we decided not to contact participants from that site. No Arabic speaking intervention participants were interviewed due to a lack of access to an Arabic speaking person who could conduct the interviews. As the intervention was delivered by persons outside of the research team, the intervention participants did not have any relationship with the researchers prior to, during, or after the research.

##### Staff Interviews

In total, nine staff members led the interventions, of which six had the role of the group leader and three the role of interpreter (two interpreting between Dari and Swedish, one between Arabic and Swedish.) The interpreters held critical roles in developing the intervention and checking its cultural appropriateness, as well as during the actual sessions where they had the role of cultural brokers (for a definition, see Bonevski et al. 2014 [15]). Four of the group leaders were master students in the last year of the Clinical Master’s Program in Psychology, and two were licensed psychologists working at a health care center close to an asylum accommodation center. For the staff interviews, we reached out to all former group leaders and interpreters. Five of them consented to participate in the qualitative evaluation, thus constituting the final sample for the staff interviews. Of these, three had had the role of the group leader and two the role of interpreter. Two were female, and three were male. Hence, some of the staff members interviewed were former students at the researchers’ department, and some of them had been employed as interpreters. These relations include an aspect of power differences. With respect to this, the staff interviews were conducted by another psychology master student (J.H.), having no prior relationship with the participants in the staff interviews. Confidentiality—in terms of recordings only being handled by the interviewer and transcriptions being fully anonymized, as well as voluntariness in participating and answering questions—was emphasized in all contacts between interviewer and participants.

### 2.3. Procedure

Between November 2017 and September 2019, six intervention groups were held at two different asylum accommodation centers in Sweden. At the first center, there was one intervention group in Dari and one in Arabic. These were open to both men and women. At the second center, there were three intervention groups in Dari and one in Arabic. One intervention group included only Dari-speaking women, whereas the remaining three groups included only men. The intervention groups were held in Swedish together with an Arabic or Dari speaking interpreter. Thus, regardless of nationality, participants speaking any of the languages could enter the group. Group size varied between 2 and 8 participants.

#### 2.3.1. Description of the Intervention

The intervention AMIN is a group psycho-educational program, a course about adaptive mental health strategies. Participants learn about how they can take care of themselves psychologically, even while living under difficult circumstances, as in an asylum accommodation center.

Some of the authors were involved in developing and designing AMIN. It was initially tested with one Dari-speaking group and one Arabic-speaking group. After this initial testing, some adjustments were made. For example, one session was divided into two. Culturally inappropriate material was removed (e.g., a lion meant to illustrate anxiety was offensive to the Syrian participants since the Arabic word for “the lion” is “al assad”, i.e., the name of the Syrian president). The trauma session was redesigned to be less intrusive. For example, an exposure oriented exercise was replaced with the “Safe Place technique” [16], which has shown helpful in treatment for young refugees [8], and the vignette was rewritten. The final version of the intervention consisted of six sessions. An introduction meeting was followed by four sessions addressing specific themes: 1. Sleep disturbances, 2. Depressed mood, 3. Worrying, and 4. Traumatic experiences. The last session was devoted to discussing relapse prevention and providing information about how to seek further help if needed (see Table 1 for an overview of the content). The included strategies regarding sleep, anxiety and depressed mood are commonly used in cognitive behavioral therapies for each problem area [17,18,19,20]. These strategies were chosen as they had the potential to be easily learned in a brief period of time and in a group context. In order to promote social cohesion and self-efficacy, the participants were encouraged to discuss with each other and contribute with their own suggestions on strategies to handle the problem at stake.

Each session lasted about 3 h. During each theme session, information was provided regarding the presented theme, and homework from the previous session was reviewed. The participants listened to a vignette in which an actor, supposedly suffering from the aforementioned problem, described his/her experiences. The participants then discussed if they recognized these experiences. Thereafter, the group leader introduced an exercise in which a strategy to handle the problem at stake was presented. This was followed up with another vignette, in which the actor was feeling better and talked about strategies that he/she had learned and used. The participants were then encouraged to contribute with examples of strategies that they had tried themselves. Before the session ended, each participant chose one strategy that he/she agreed to try out during the following week.

#### 2.3.2. Training of Staff

The intervention groups delivered at the first asylum accommodation center were led by psychology master students with ongoing supervision by licensed psychologists throughout the intervention. Their training consisted of three sessions, spread over a month. It started with a six-hour session on psychological assessment and treatment with refugees. This was followed by a three-hour review of each intervention session’s content and theoretical underpinnings. Finally, a five-hour workshop was held. Here, the students had prepared different sessions, and in the workshop, they practiced providing psychoeducation and leading exercises together with the interpreters. The intervention groups at the second asylum accommodation center were led by licensed psychologists, and their training was briefer, consisting only of a 3-h review of the manual and information on how to administer the data collection. The licensed psychologists did not receive regular supervision but were in contact with the research team when needed.

#### 2.3.3. Quantitative Evaluation Procedure

At both asylum accommodation centers, information about the intervention was provided by project personnel, volunteers and staff. Potential intervention participants were invited to the first introductory meeting. The meeting was initiated with verbal and written information about participation in the intervention and in the research study, in Arabic or Dari, followed by answering any questions. If individuals wanted to participate in the study, they gave an active digital consent on an iPad, on which they also completed the pre-assessment. The intervention was thereafter delivered for five consecutive weeks. Post assessment was conducted in conjunction with the last intervention session. All quantitative data were collected through the online software system, Qualtrics (Qualtrics, Provo, UT, USA), through iPads (Apple Inc., Cupertino, CA, USA).

#### 2.3.4. Qualitative Evaluation Procedure

##### Intervention Participant Interviews

The intervention participant interviews were performed via phone by two different persons fluent in Dari. All interviews were recorded. Before starting the interview, the interviewer informed participants about the interview procedure and answered any questions. Thereafter the interviewer asked if the participant consented to the interview procedure and noted the response. A semi-structured interview guide was used. The questions regarded the intervention participants’ opinions about the intervention, both positive and negative if they thought that there was something missing, and the opposite, if they found parts of the intervention superfluous. Participants were asked whether they would recommend this intervention to a friend and also about possible reasons to decline an offer to participate in the intervention. Interviews were recorded and transcribed verbatim in Dari by the person who had conducted the interview. Two interviews were translated directly into English by the person who had conducted the interviews. However, one of the interviewers did not speak English. Thus, three of the interviews were first translated from Dari to Swedish by this interviewer and thereafter from Swedish to English by the first author of this paper (A.L.).

##### Staff Interviews

The staff interviews were conducted with the same procedure, as described above, by a psychology master student (J.H.), using a semi-structured interview guide. The questions regarded the staff’s opinions on the intervention, in terms of how understandable it was to the intervention participants, how clinically meaningful they as group leaders perceived it to be, and how feasible it was to deliver. All interviews were recorded and then transcribed verbatim by the interviewer. The first author of this paper (A.L.) translated the quotes from Swedish to English.

### 2.4. Assessment

To assess the potential effects on mental health, as well as acceptability and feasibility of the intervention, both quantitative measures and semi-structured interviews with intervention participants and staff members were used. The quantitative assessment of feasibility was based on retention and attendance rates (see Table 2, implementation matrix, for an overview of the strategies used to assess each research question.).

#### Quantitative Outcome Measures

Quantitative data assessing the potential effectiveness of the intervention was gathered using self-assessment scales pre- and post the intervention. To assess the overall effects of the intervention on symptoms of distress, we used the refugee health screener (RHS), 13 item version [21]. The RHS is designed to screen for symptoms of anxiety, depression, and PTSD among refugees, and it ranges from 0–52. Scores between ≥ 11 < 18 indicate mild symptoms, ≥ 18 < 25 indicates moderate symptoms, and ≥ 25 indicates severe symptoms [22]. To assess potential effects on sleeping problems, we used the Insomnia Severity Index [23], which ranges between 0 and 28 points. Scores 0–7 indicate no clinically significant insomnia, scores between 8 and 14 indicate subthreshold insomnia, scores between 15 and 21 clinical insomnia of moderate severity, and scores between 22 and 28 severe clinical insomnia. To assess potential effects on quality of life (QOL), we used the World Health Organization Quality of Life—brief version (WHOQOL-Bref) [24]. The WHOQOL-BREF contains four different domains: physical, psychological, social and environmental health and quality of life. Raw scores are converted to domain scores ranging from 4 to 20. Skevington and colleagues estimated that the mean domain scores in a sample of 11,830 adults from 23 different countries were 16.2 (*SD =* 2.9) for physical QOL, 15.0 (*SD =* 2.8) for Psychological QOL, 14.3 (*SD =* 3.2) for social QOL and 13.5 (*SD =* 2.6) for environmental QOL [25]. A score of 12 has been defined as a midpoint where the quality of life is judged to be neither good nor bad. To assess whether the intervention could reduce the catastrophizing of symptoms, we used the symptom-catastrophizing scale (SCS) [26]. The SCS is scored from 0–2, contains seven questions and thus ranges from 0–14 points. Among depressed patients [26], a pretreatment mean score of 10.7 (*SD =* 2.6) has been found. In a sample of patients with PTSD [27], the mean score was 10.3 (*SD =* 3.0).

### 2.5. Data Preparation and Analysis

#### 2.5.1. Quantitative Analysis

The statistical analyses were carried out using IBM SPSS Statistics for Windows (version 25, IBM Corp, Armonk, NY, USA). Due to the low sample size, we used the Wilcoxon signed-rank test to assess the difference pre and post the intervention on all included measures. Effect sizes were calculated using Pearson’s correlation coefficient *r.*

#### 2.5.2. Qualitative Analysis

The recorded interviews were transcribed verbatim. Interviews from the intervention participant and the staff were analyzed separately using thematic analysis [28]. The transcriptions were read through several times. Quotes relevant to the aim of the study were marked and condensed to meaning units, which were extracted to tables and labeled with a code. The codes were then sorted into possible themes and sub-themes. To ensure that the findings and the raw data corresponded, the final themes and sub-themes were named and checked against the transcribed interviews.

#### 2.5.3. Integration of Quantitative and Qualitative Results

When both the quantitative and the qualitative data were analyzed, the entire material was reviewed again. The material was now approached in a deductive manner, based on the research questions regarding potential effectiveness, acceptability and feasibility of the intervention. In the first step, the two qualitative datasets were compared. Themes and quotes related to potential effectiveness, acceptability, and feasibility were extracted from both datasets and gathered in a new document. The extracted quotes were backtracked to the transcriptions to make sure that they were representative of the raw data and not taken out of context. In the staff material, this process resulted in one original theme being split into two, whereas one part of the theme was assessed as belonging to acceptability and the other part to feasibility. Themes and sub-themes used to assess each research question are displayed in Table 2, implementation matrix. The full transcriptions of both the intervention participant interviews and staff interviews were reviewed once more in order to make sure that no important information regarding effectiveness, acceptability or feasibility was missing. After this, the quantitative and qualitative results were merged. In this process, it was decided that physical and psychological QOL contributed more to the understanding of the intervention’s acceptability than to its potential effectiveness. The quantitative and the qualitative results were compared for each research question in order to discover overarching common themes as well as points of divergence.

## 3. Results

### 3.1. Summary of Quantitative Results

In sum, there were significant reductions in psychological distress and insomnia between the pre- and post-assessments. Furthermore, an increase could be seen in the physical quality of life. These differences were of medium-sized effects (*r* from 0.35 to 0.42). No other included measures showed any significant difference. A full display of the quantitative results can be found in the mixed methods result section.

### 3.2. Initial Qualitative Findings

#### 3.2.1. Intervention Participant Interviews

Five themes emerged during the intervention participant interviews. These were “an overall positive impression”, “continual use of strategies”, “pros and cons of being in a group”, “mental health after finishing the course,” and “contextual factors affecting wellbeing”. Since the material was rather brief, no further division into subthemes was made.

#### 3.2.2. Staff Interviews

Four themes emerged from the staff interviews. These were “facing a broad range of needs”, “perceived benefits of the intervention”, “who can deliver this intervention?” and “the importance of sharing and of relationships”. These themes were further divided into subthemes, see Table 3.

### 3.3. Mixed Methods Results

How the themes from the intervention participant interviews and the staff interviews were sorted into the broad categories “potential effectiveness” and “acceptability” can be seen in Table 2, implementation matrix. While integrating the results, four sub-themes were formed under “potential effectiveness”: “prevention is not enough”, “was a potentially positive effect caused by normalization?”, “the importance of human encounters” and “the housing conditions and the asylum process influenced the participants’ mental health”. Under the theme “acceptability”, the sub-theme “not all sessions were equally accepted” was formed. Under the theme “feasibility”, the sub-themes “attendance”, “the non-negligible influence of contextual factors”, “a mixed group” and “who can deliver this intervention?” were formed. In Table 4, Table 5 and Table 6, the integrated qualitative and quantitative results on potential effectiveness, acceptability and feasibility are shown. The findings are presented in conjunction with quantitative and qualitative results for a clear display of where these results converge or diverge. Exhaustive narrative reports on each mixed-method finding can be found below tables.

#### 3.3.1. Potential Effectiveness

The intervention seemed to hold a potential for reducing symptoms of distress and insomnia, although the participants suffered from more severe distress than expected. The staff expressed the idea that a possible effect was caused by the normalization of symptoms. However, this was not supported by quantitative measures, nor was the perception of the importance of human encounters. The asylum process, including the conditions at the asylum accommodation centers, seems to have affected the intervention participants’ mental health and made it difficult for them to apply some of the suggested strategies. See Table 4 for a joint display of quantitative and qualitative results concerning potential effectiveness.

The median score of distress pre-assessment was in the range of severe distress (28.0, MAD 11.0), whereas the post-assessment median score was in the range of moderate distress (21.0, MAD 9.0). The reported levels of insomnia were also reduced from a pre median score of 16.0 (6.0), indicating clinical insomnia of moderate severity, to a post-median score of 11.0 (5.0), indicating subclinical symptoms of insomnia. Participants also reported feeling better after the course, mainly related to being more social and active.

*Before I could not speak with people, I was just thinking alone, my head would grow bigger and bigger and I wanted to run away from public spaces. But now I am participating in such courses, which helps a lot. Now I talk with people and I participate in gatherings.*

There were also reports of the course opening the door to individual psychological treatment at the primary care center. However, some participants reported that the initial wellbeing experienced during the course declined rather fast, and others reported current mental ill health.

*… I was feeling better when I went to that course. They were talking with us and we could talk about things. I also had another doctor in Red Cross I could talk with. Now both of them are finished, and after the courses ended, my moral came down.*

The staff was cautious not to speculate about the possible effects on participant mental health, “Only the post-assessment can say anything about this”, one staff member stated. However, the general opinion among staff was that the course was useful to the participants. “After the course, they learned how to handle their symptoms… so in that way the course was really good and useful” one staff member said.

##### Prevention Is Not Enough

The staff expressed concerns regarding the intervention’s preventative and psycho-educational approach and suggested that this may not be sufficient for the participants.

*But if you don’t eat, if you don’t sleep... then it is quite useless to learn the depression model… at least that’s what I think. That maybe you have to start with these basic things, and this group of patients was so impaired that it felt like that was where we were at.*

The quantitative data supports the notion that participants were in need of more than the course could provide, such as specialized care. The median score on the RHS pre-intervention was 28.0 (MAD 11.0), which is above cutoff 25, indicating severe distress [22].

##### Was a Potentially Positive Effect Caused by Normalization?

In the staff interviews, the perception that the course could help normalize symptoms and experiences emerged. This was thought to increase the participants’ willingness to seek help in the future.

*… with the fact that one may normalize these symptoms, perhaps the… that patients are more willing to seek help later on. (…), like ‘There is a word for what I am experiencing and there are people who are actually working with it in health care, people who could actually help me’.*

When the participants were asked how they viewed their mental health and their symptoms before and after taking part in the intervention, no clear answers were received. The staff’s perception was not supported by the results from the symptom-catastrophizing scale, with no significant change between the pre (Md 7.5, MAD 2.5) and post-assessment (Md 7.0, MAD 4.0).

##### The Importance of Human Encounters

Intervention participants expressed that the group format was something positive, that it relieves feelings of solitude and allowed suggestions and advice to be shared: “It was good (to be in a group), I liked it, we met and gave each other comments and suggestions…” one intervention participant said. However, there were different opinions regarding how freely participants could express themselves. Some found that this was possible thanks to common rules of non-disclosure, but there were also expressions of some issues being hard to discuss in a group: “I was not that comfortable being in a group, it is not possible to share certain personal problems in front of an entire group”, one intervention participant stated.

The staff regarded the relief of putting words into feelings and sharing experiences with other asylum seekers as an important part of the intervention. “To talk in a safe space, to share experiences and perceptions, to put words on it maybe, that was important to them”, one staff member said. However, the quantitative measure “social quality of life” showed no significant difference between the pre (Md 10.7, MAD 2.7) and post-assessment (Md 12.0 MAD 2.7).

##### The housing conditions and the asylum process influenced the participants’ mental health

In the participant interviews, the difficult context of seeking asylum and living in an asylum accommodation center was frequently mentioned as something that affected mental health negatively.

*We got a negative response [to the application for residence permit] one year and eight months ago… (I worry) about the negative response… for three years we have been living in this camp in one room, which is very difficult for us. Then what to do, I don’t know, whom to talk to about our pains?*

The staff also emphasized how deeply the asylum process and the conditions at the centers affected the participants’ mental health.

*What they need is a residence permit and somewhere to live that is not in an asylum accommodation, so they don’t have to wait in line to get food, share their bathroom with 20 others. There’s a lot of that stuff that I think might be a little more urgent to them than attending a depression treatment.*

The median score on the environmental domain of quality of life was low pre-intervention (Md 10.5, MAD 2.0) and remained unchanged at the post-assessment (Md 10.0, MAD 2.0), underscoring the importance of the context and the negative influence of the housing situation.

#### 3.3.2. Acceptability

The intervention generally seems to have been accepted by the intervention participants, but in the staff interviews, large differences were found between the various themes. The notion of differences in acceptability between the sessions was supported by the results of the outcome measures. In Table 5, a joint display of the results regarding acceptability is shown.

The intervention participant interviews pointed to a general acceptance of the content and structure of the intervention. They stated that the course was valuable, that it was good for people suffering from mental ill-health, and for those feeling alone and longing for their families. There were no negative comments, although verbally probed for.

*It [the course] is very good for those who do not feel well psychologically, they will get a positive result from attending. One side: people have problems in Afghanistan, on the other side they are worried and unemployed in Sweden. … Most have been harassed and suffered many problems on their way here. Undoubtedly, this course will be helpful to them.*

The intervention participants also unanimously stated that if the course were to be given again, they would recommend it to a friend: “If a friend of mine wasn’t feeling well psychologically I would recommend him to take this course, 100%….” one participant said. The intervention generally seems to have been acceptable to the staff as well, “It really addressed themes that were relevant to the target group I think, in a manner that was… it felt right, not too complicated…” one staff member said. However, the staff also expressed that it was difficult to maintain focus on the psychoeducation. The intervention participants had a strong need to talk about previous and current hardships, something that may be interpreted as a lack of acceptance of the intervention’s structured, educational format.

*I tried to do the psychoeducation, etc., and I tried to make everything pedagogically and so on, but then it was like, the need to talk about the current situation and how hard everything was, was stronger. (…) They were somewhere else in their minds and perhaps they saw me a little more as someone who might be able to help on a more concrete level.*

##### Not All Sessions Were Equally Accepted

From the staff’s point of view, the intervention sessions varied in acceptability. The staff perceived parts of the intervention as difficult for the intervention participants to understand. Worry, and specifically the strategy “worry time”, was regarded as too abstract and complicated.

*There were different strategies there* [in the worry session] *and one of them was about “worry time”, that you could decide a moment or a specific time to worry, this was incomprehensible to many.*

Unlike worry time, the concrete, health-promoting strategies were easily absorbed. Behavioral activation and “The Tower”, an exercise where participants were asked to estimate their energy level before and after a playful group activity, were highlighted as examples of particularly appreciated elements. This exercise seemed to be a good way to connect theory and practice by showing how activity affects mood. In addition, the tower-exercise contributed to a good atmosphere, which was perceived as a valuable aspect in itself.

*One thing I remember that they were really helped by was this exercise that the interpreter had invented, where you were supposed to build towers, and it was like this how... to estimate how you feel before and estimate how you feel afterwards to show that what we do also affects how we feel. It was really great and there... it was a real eye-opener to them.*

When the participants were asked if they continued using any strategies they had learned during the course, activation was often mentioned: “For example: I do sports, relax, focus on myself before going to sleep, spend time with friends. You should not think too much”, one intervention participant said, which makes it plausible that the participants were susceptible to this part of the intervention. Several intervention participants expressed that they learned not to think too much during the course. This implicates that the idea of restricting worry did catch on, although the theoretical basis and the practical implication of this may be a bit less straightforward.

*Some participants tried “Worry time”, it’s unclear how they interpreted it, I’m not sure it was “by the book”, but they sat there for a while and… *[you should] *not take the worry time when you feel like it, then it’s just worrying. Worry is a bit abstract...*

In line with the interviews pointing at the behavioral activation as an important part of the intervention, the quantitative results showed an increase in the physical domain of quality of life Md 11.7, MAD 1.7 to Md 12.6, MAD 2.3, see Table 6). The psychological domain of quality of life did not change post-intervention (Pre Md 10.7, MAD 2.7, Post Md 12.7, MAD 3.3). Although not explicitly targeting worry, this could perhaps be regarded as a support for the notion of the abstract aspects of the intervention as less effective.

#### 3.3.3. Feasibility

Feasibility was assessed using quantitative data on attendance and attrition and the staff interviews. The staff raised the notion of the impact of contextual factors and difficulties associated with providing intervention to a population consisting of individuals with different backgrounds and conditions but also highlighted aspects enhancing feasibility.

##### Attendance

On average, each intervention participant attended 4.5 (*SD =* 1.4) out of 6 possible sessions. Each session was in total attended by between 15 and 19 participants (i.e., 60–75%). One participant (4.0% of the 25 participants) attended 2 sessions, 3 participants (12,0%) attended 3 sessions, 8 participants (32.0%) attended 4 sessions, 6 (24.0%) participants attended 5 sessions and 6 participants (24.0%) attended 6 session, see Table 6. Data regarding attendance are lacking for one of the intervention participants. Non-attendance was generally attributed to forgetfulness: “I think I could not attend the course for two weeks, as I forgot that and one other week I don’t know what happened, I could not attend, but the rest I attended”, one intervention participant explained.

##### The Non-Negligible Influence of Contextual Factors

Apart from affecting the intervention participants’ mental health in a negative manner, the unsafe housing situation also reduced the possibility to participate in the course from beginning to end, as some participants were transferred or even deported during the intervention. Of the 52 intervention participants attending the information meeting and consenting to participate in the study, 17 (32.7%) dropped out. Unfortunately, the reasons were not systematically recorded, but there are some verbal accounts indicating that drop-out rates were at least partly due to migration issues.

*The atmosphere* [in the group] *was ups and downs all the time. One of the participants, who was very committed and interested in participating, was deported after the second meeting, and the others were affected by this event. But another positive event was at maybe the fourth meeting… one of the participants came with good news: that he had received a residence permit and then it was all like joy and positive emotions.*

*It is a fairly long period *[addressing the length of the intervention]* and during this time they can be forced to move at any time, if that’s what the Migration Agency decides. This was a challenge and we lost many people interested in the course because of that, they cannot choose it, they are forced to move.*

After the initial testing, where a group was held at the university, the decision was made to conduct the intervention on-site, at the asylum accommodation centers. This seems to have been a factor increasing the feasibility of delivering the intervention and the possibility for the participants to attend: “It’s good if it is here because it’s nearby, but if it takes place far from here, we cannot attend, because we don’t have bus passes for travelling”, one intervention participant stated. However, the staff was forced to travel long distances: “It was tiring to drive 2 h to get there, then 2 h to get back, plus 3 h intervention, you spent almost an entire day…” one staff member said.

##### A Mixed Population

The intervention groups consisted of individuals with different language skills, educational levels, and backgrounds, which led to different levels of understanding. To keep the information at a level suitable for all intervention participants was challenging for the staff.

*For one thing, it was a very mixed group, I don’t think there were any illiterates in the group but close to it, and then we had others who were extremely highly educated (…) It’s such a mixed population.*

By doing exercises together, the participants were actively involved, which was highlighted as a successful approach to increase understanding. Here, even participants who turned out to have insufficient language proficiency (something that eventually made them leave the group) could participate in a meaningful manner.

*We could connect the theoretical knowledge with something practical. … even if they had a hard time communicating with other group leaders... We had two participants in the group who could not speak Arabic, but here that was not an obstacle, they could communicate with their body language.*

##### Patience and Flexibility

Implementing the intervention was both energy and time-consuming, so patience and flexibility were emphasized as important skills. One staff member said, “It often took longer than you’d think. (…) Especially if there were a lot of delays, and there were, every time”. The importance of continually making adjustments was also emphasized. “I think that was the best about the entire intervention, that we adapted to the situation, I think if we would have been very strict, they would not have come the next time”, one staff member stated.

##### Experience and Education

Group leaders reported that the intervention was easy to implement, and the format was described as pedagogical and structured. The pre-intervention training also seemed to have been sufficient. Previous experience of working with the target group was beneficial. The group leaders believed that professionals without any formal training in psychology would be able to deliver the intervention, with some additional training and supervision.

*… you might need a little more education on how to detect severe mental illness… but you could use assessment instruments for that, kind of like a ‘red flag’. Maybe you don’t need any training for that, if you have the assessment instruments and a supervisor you can ask.*

## 4. Discussion

This paper constitutes an initial evaluation of a psycho-educational group intervention provided at asylum accommodation centers in Sweden. Using a combination of quantitative assessments and interviews with both participants and staff members, a preliminary picture of this intervention’s benefits and improvement needs could be drawn.

### 4.1. Potential Effectiveness

According to both quantitative and qualitative data, this intervention seems to hold the potential for a positive effect on participant mental health. This general finding is in line with recent reports [29,30] and supports the notion that it is indeed meaningful to provide psychosocial interventions to individuals still in the asylum process.

Weine [31] discusses the importance of timing when planning interventions for refugees. Some intervention participants reported that they had already been in Sweden for several years when attending the intervention. This intervention was designed to be an early intervention, and perhaps it would be more effective and suitable if provided earlier on.

In the staff interviews, the idea that a possible positive effect could be due to a normalization of symptoms arose. Psychoeducation can provide an individual with an increased sense of control over his/her symptoms [32], which in turn could decrease the additional burden a person may experience from worrying about the symptoms themselves. However, no change could be seen in the symptom-catastrophizing scale, indicating that this was not the case here. The pre median score on the SCS in this study was closer to the post mean score around 6.5 previously found [26,27]. Hence, the pre score of this study may not have been elevated at all. Another aspect to consider is that the SCS had not previously been used in refugee populations and may lack transcultural validity. What normalization actually means in this context could also be debated. Here, it does not seem to mean that the symptoms are regarded as normal; however, it could also be that meeting others with similar experiences could provide some kind of relief. In that sense, a possible positive effect could be related to an increased sense of social support, something that has previously been found to be utterly important for refugee mental health [33]. The importance of social support was indeed raised both by intervention participants and staff; however, no effect was seen in the quantitative measure of social QOL. This could indicate that social support should be emphasized in a more explicit manner in the intervention. It could, however, also be that the social domain of the WHOQOL-BREF was insufficient in capturing a possible effect. This domain consists of only three items, one being “how satisfied are you with your sex life”, something that was not targeted by the intervention. In a future trial, a more extensive instrument focusing on social support could thus be added.

The post migratory stressors in terms of harsh living conditions and insecurity regarding the result of the asylum application affected the intervention participants’ mental health negatively, and the pre-intervention scores on WHOQOL-BREF were all lower than the scores previously been found in the general population [25]. This is in line with previous research showing a clear connection between post migratory stressors and mental ill-health among asylum seekers [34,35]. These results may also implicate that a stronger emphasis on support and guidance regarding the current situation could be beneficial for the intervention participants’ mental health.

### 4.2. Acceptability

Regarding acceptability, the content and the form generally seem to have been acceptable to the participants. In the staff interviews, however, it was made clear that there were large differences between different elements in the intervention. The worry session was perceived as the most problematic. One interpretation of this result is that the concept of “worry” and the strategies to handle it is more culturally specific than the other strategies. In a review on anxiety disorders and culture, Lewis-Fernández et al. [36] discuss how patients from many non-western countries endorse somatic symptoms as key aspects of worry, whereas patients of European descent predominantly presents psychological and cognitive symptoms of worry. These results could thus indicate that this session requires further cultural adaptation.

### 4.3. Feasibility

In this study, 32.7 % of the participants dropped out for unknown reasons. In a meta-analysis on drop-out from Cognitive Behavioral Therapy [37], the average weighted drop-out during treatment was 26.2%. Since the current study took place under extraordinary circumstances during the asylum process at an asylum accommodation center, a higher drop-out rate could be expected. Hence, these numbers may be regarded as acceptable.

In this study, the attendance rate (75.7% or 4.5 of 6 possible sessions) was lower than in a study evaluating an intervention similar to AMIN: Problem Management Plus [38], where the attendance rate was 84%; 4.2 (*SD =* 1.7) sessions out of 5 possible. Again, due to the specific circumstances of our intervention, a lower rate could be acceptable. Weine [31] states that while developing preventative interventions for refugee families, one must put much effort in engagement activities (to get people to join and keep attending) and that it is important to maximize the convenience of the intervention for the participants. This intervention was delivered at the asylum accommodation centers and at a time of the day that was perceived as beneficial for the intervention participants. More on, the cultural brokers sent reminders to the intervention participants. However, the need for further adjustments or assistance to raise attendance is something that could be investigated by interviewing participants before a larger trial is conducted.

Regarding feasibility in delivery, some difficulties were brought to attention in the staff interviews. One regarded the contextual factors. Albeit difficult to change, one adjustment could be to shorten the length of the intervention, thus reducing the risk of participants having to leave in the middle of it. A review of the characteristics of successful interventions for refugees [31] emphasizes the importance of the intervention being focused on time. As one staff member expressed, six weeks may be too long. Another difficulty was the inherent difference between the participants, in everything from educational level to mental health problems. This confirms results from previous studies showing that heterogeneity among refugees makes it difficult to design interventions that suit the entire group [30,39,40]. The intervention was provided in the participants’ native language, and it was intended to be conducted with relatively culturally homogenous groups, two factors that have been found to increase the effectiveness of interventions [41]. Although this may be positive in terms of effectiveness, it was ethically problematic since asylum seekers speaking less common languages were not offered participation. At the first center, it was also difficult to recruit enough participants to be able to construct homogenous groups in Arabic.

The staff expressed that the manual was easy to follow and that the intervention could, without extensive training or supervision, probably be delivered by individuals with different professional backgrounds. In this study, two different levels of training were provided. Both seemed to be adequate, considering the different levels of experience of the group leaders (master students vs. licensed psychologists). Thus, if the intervention were to be delivered by individuals without formal training in psychology, the more extensive training model ought to be applied, alongside continuous supervision. With these measures taken, this intervention could be possible to implement in a stepped care model, as described by IASC [6].

### 4.4. Methodological Discussion

When interpreting the results of this study, some methodological limitations should be considered. One obvious limitation in determining potential effectiveness concerns the design of this study. Without randomization or a control group, it is not possible to distinguish the potential effects of the intervention from other confounding factors. We initially planned to randomize participants to a control intervention, but we soon found out that this was not feasible nor ethical among individuals living at the same accommodation center. However, in a future trial, cluster randomization should be considered. Larger sample size is also needed to gain enough power to detect changes caused by the intervention. In this study, no follow-up assessment was conducted. This should, of course, be included in a future trial, especially since one intervention participant indicated that her wellbeing had deteriorated after finishing the course.

Regarding the qualitative assessment, the staff interviews provided a rather rich and nuanced material, despite the fact that some of the interviews took place after almost two years. The fact that the staff provided both positive and negative opinions on the intervention indicates that they felt comfortable in sharing different types of experiences, regardless of prior relationships with the researchers.

The intervention participant interviews, on the other hand, were all rather brief and unbalanced in their positive expressions regarding the intervention. It could not be ruled out that power relations between the intervention participants and the researchers, in combination with the intervention participants’ utterly vulnerable position as asylum seekers in a foreign country, made it difficult for them to freely express negative opinions. The fact that the interviews were carried out by a fellow countryman they had never met before, in the participants’ native language, does not seem to have made a difference.

Another limitation regarding the intervention participant interviews stems from the fact that they were translated, a procedure that entails a risk of nuances and meanings getting lost. Some of the interviews were even translated twice: first from Dari to Swedish, and then from Swedish to English. This could put the reliability of the qualitative analysis into question. However, we do believe that the essence of the interviews was captured adequately. Due to difficulties in the process of hiring a Dari-speaking interviewer, the intervention participant interviews could not take place directly after the intervention, which may have affected the quality of the interviews as well as the final sample size of this sub-study. The selection of participants to interview was further limited to the Dari speaking participants since no Arabic speaking staff member was available to perform interviews. Ideally, a maximum variation sampling strategy would have been used in the qualitative sub-studies instead of convenience sampling. Since the intervention participants, as was pointed out in the staff interviews, was a mixed group, it would have been beneficial to interview intervention participants from all ages, languages and sexes.

## 5. Conclusions

This mixed-method study provided a preliminary evaluation of a psycho-educational intervention provided at asylum accommodation centers in Sweden. These preliminary results are promising in terms of potential effectiveness, acceptability and feasibility. However, the results also indicate that the intervention could benefit from some adjustments, particularly in the session regarding worry.

## Figures and Tables

**Table 1:** Overview of the intervention.

Session	Description of Content	Strategies Taught	Exercise
1.	Presentation, overview of course, pre-assessment		
2.	Sleep	Sleep hygiene, stimulus control	Relaxation
3.	Depression	Behavioral activation	Assess energy level before and after a collaborative game
4.	Worry	Worry time, problem-solving	Sort worry type, role-play problem-solving
5.	Trauma	Recall coping strategies	Safe place
6.	Relapse prevention	When to seek help	Write down things to keep doing to feel well

**Table 2:** Implementation matrix.

Research Question	Strategy	Sample	Time for Data Collection	Analysis
Potential Effectiveness?	QUANT: self-assessment scales: The refugee health screener (RHS) Insomnia severity index (ISI) World Health Organization Quality of Life—brief version (WHOQOL-BREF); social and environmental QOL symptom catastrophizing scale (SCS)	25 individuals living in an asylum accommodation center in Sweden, participating in the intervention	Pre-assessment at first intervention session and post-assessment at last intervention session	Wilcoxon signed-rank test to assess pre and post difference, Pearson’s r for effect sizes
	QUAL: participant interviews, themes: “mental health after the intervention”, “pros and cons of being in a group”, “contextual factors affecting wellbeing.”	Convenience sample of five Dari speaking intervention participants	1–6 months post-intervention	Thematic analysis
	QUAL: staff interviews, sub-themes: “contextual factors influencing the outcome”, “the need to talk about previous and current hardships”, “in need of proper treatment, not just prevention”, “to share difficulties”, “human encounters” and “growing relationships between and among participants and staff”	Convenience sample of five former staff members	6 months to 2 years post-intervention	Thematic analysis
Acceptability?	QUANT: WHOQOL-BREF; physical and psychological QOL	25 individuals living in an asylum accommodation center in Sweden participating in the intervention	Collected during the intervention	Wilcoxon signed-rank test to assess pre and post difference, Pearson’s r for effect sizes
	QUAL: participant interviews, themes: “an overall positive impression”, “continual use of strategies”	Convenience sample of 5 Dari speaking participants	1–6 months post-intervention	Thematic analysis
	QUAL: Staff interviews, sub-themes: “a heterogeneous group”, “from the abstract to the concrete”	Convenience sample of five former staff members	6 months to 2 years post-intervention	Thematic analysis
Feasibility?	QUAL: Staff interviews, sub-themes: “contextual factors affecting outcome”, “patience and flexibility”, “previous experience and education”	Convenience sample of five former staff members	6 months to 2 years post-intervention	Thematic analysis
	QUANT: drop-out and attendance rates	The 52 individuals attending the first meeting vs. the 25 attending the last meeting, number of sessions visited by each participant	At the first and last intervention session	Descriptive analysis

Note: quant = quantitative information; qual = qualitative information.

**Table 3:** Themes ad sub-themes from the staff interviews.

Theme	Sub Theme
Facing a broad range of needs	Contextual factors influencing the outcome The need to talk about current and previous hardships In need of proper treatment, not just prevention
Perceived benefits of the intervention	A heterogeneous group From the abstract to the concrete
Who can deliver this intervention?	Patience and flexibility Previous experience and education
The importance of relationships	To share difficulties Human encounters and growing relationships between and among participants and staff

**Table 4:** Joint display of results on potential effectiveness.

		Qualitative Support?	
Finding	Quantitative Support?	Participant Interview	Staff Interview
Potential effectiveness	YES: RHS (*n* = 25) pre 28.0 (11.0), post 21.0 (9.0) *Z* = −2.50, *p* = 0.012, *r* = 0.35 ISI (*n* = 25): pre 16.0 (6.0), post 11.0 (5.0) *Z* = −2.55, *p* = 0.011, *r* = 0.36	YES: *I was feeling better when I went to that course*	YES: *After the course, they learned how to handle their symptoms.*
Prevention is not enough	YES: PRE RHS: Md 28.0 (11.0), above cutoff 25 for severe distress	No participant sayings on this	YES: *But if you don’t eat, if you don’t sleep. then it is quite useless to learn the depression model…*
Was a potentially positive effect caused by normalization?	NO: SCS (*n* = 20): pre 7.5 (2.5), post 7.0 (4.0) *Z* = −1.02, *p* = 0.310, *r* = 0.16	No participant saying on this	YES: *… one may normalize these symptoms…*
The importance of human encounters	NO: Social QOL (*n* = 19): pre 10.7 (2.7), post 12.0 (2.7) *Z* = −0.73, *p* = 0.467, *r* = 0.12	*Good to be in a group…,* but also *it is not possible to share certain things in a group…*	YES: *to share experiences and perceptions (…) that was important to them*
The housing conditions and the asylum process influenced the participants’ mental health	YES: Environmental QOL (*n* = 19) pre: 10.5 (2.0), post 10.0 (2.0) *Z* = −0.81, *p* = 0.419, *r* = 0.13	YES: *We got a negative [response to the application for residence permit] one year and eight months ago (…) for three years we have been living in this camp in one room…*	YES: *What they need is a residence permit and somewhere to live that is not in an asylum accommodation…*

Note: RHS = the refugee health screener, ISI = insomnia severity index, SCS = symptom severity scale, QOL = quality of life.

**Table 5:** Joint display of results on acceptability.

Finding	Quantitative Support?	Qualitative Support?	
		Participant Interview	Staff Interview
General:	No quantitative data	YES: *If a friend of mine wasn’t feeling well psychologically I would recommend him to take this course….*	YES: *It addressed themes that were relevant to the target group in a manner that was not too complicated…*
Not all sessions were equally accepted	YES: physical QOL (*n* = 18), Pre 11.7 (1.7), Post 12.6 (2.3), *Z* = −2.50, *p* = 0.013, *r* = 0.42, but: NO: psychological QOL (*n* =19), Pre 10.7 (2.7), Post 12.7 (3.3), *Z* = −0.83, *p* = 0.407, *r* = 0.13	YES: *For example: I do sports, relax, focus on myself before going to sleep, spend time with friends. You shouldn’t think too much*	YES: *… that what we do also affects how we feel (...) it was a real eye-opener to them.* NO: *…a specific time to worry… that was incomprehensible to many.*

Note. QOL = quality of life.

**Table 6:** Joint display of results regarding feasibility.

Finding	Quantitative Support?	Qualitative Support?	
		Participant Interview	Staff Interview
Attendance	Each participant attended an average of 4.5 (*SD* = 1.4) of 6 sessions	No participant statement on this.	No staff statement on this.
The non-negligible influence of contextual factors	17 participants (32.7%) dropped out for unknown reasons.		*One of the participants, who was very committed and interested in participating, was deported after the second meeting…*
A mixed population			*I don’t think there were any illiterates in the group but close to it, and then we had others who were extremely highly educated…*
Patience and flexibility			*I think that was the best about the entire intervention, that we adapted to the situation*
Experience and education			*… you might need a little more education on how to detect severe mental illness… but maybe you don’t need any training for that, if you have the assessment instruments and a supervisor you can ask.*
